# Megakaryocytes contain extranuclear histones and may be a source of platelet-associated histones during sepsis

**DOI:** 10.1038/s41598-020-61309-3

**Published:** 2020-03-12

**Authors:** Galit H. Frydman, Shannon N. Tessier, Keith H. K. Wong, Charles R. Vanderburg, James G. Fox, Mehmet Toner, Ronald G. Tompkins, Daniel Irimia

**Affiliations:** 1 Division of Comparative Medicine and Department of Biological Engineering, Massachusetts Institute of Technology, Cambridge, Massachusetts United States of America; 2 BioMEMS Resource Center, Center for Engineering in Medicine, and Center for Surgery, Innovation and Bioengineering, Department of Surgery, Massachusetts General Hospital, Boston, Massachusetts United States of America; 3 Harvard Neurodiscovery Center, Harvard Medical School, Boston, Massachusetts United States of America

**Keywords:** Coagulation system, Haematopoietic stem cells

## Abstract

Histones are typically located within the intracellular compartment, and more specifically, within the nucleus. When histones are located within the extracellular compartment, they change roles and become damage-associated molecular patterns (DAMPs), promoting inflammation and coagulation. Patients with sepsis have increased levels of extracellular histones, which have been shown to correlate with poor prognosis and the development of sepsis-related sequelae, such as end-organ damage. Until now, neutrophils were assumed to be the primary source of circulating histones during sepsis. In this paper, we show that megakaryocytes contain extranuclear histones and transfer histones to their platelet progeny. Upon examination of isolated platelets from patients with sepsis, we identified that patients with sepsis have increased amounts of platelet-associated histones (PAHs), which appear to be correlated with the type of infection. Taken together, these results suggest that megakaryocytes and platelets may be a source of circulating histones during sepsis and should be further explored.

## Introduction

Traditionally, histones have been recognized as a functional ‘glue’ for genetic material, wherein core histones (H2A, H2B, H3, and H4) form octamers and linker histones (H1 and H5) hold DNA together in specific conformations. Specific histone post-translational modifications (PTMs) also directs site-specific activation or silencing of transcription^1^. While histones are typically located within the nucleus of the cell, they can also be located within the cytoplasm, the cell membrane, or can even be extracellular^2^. When histones are located outside of the cell they can change roles and serve as damage-associated molecular patterns (DAMPs) and be dangerously proinflammatory and cytotoxic^2,3^. Extracellular histones have been shown to be associated with life-threatening inflammatory conditions, such as sepsis and its’ secondary complications, including acute respiratory distress syndrome (ARDS) and acute kidney injury (AKI)^4–9^. Histones can also induce profound thrombocytopenia via platelet activation and aggregation; in fact, circulating histone levels in Intensive Care Unit (ICU) patients has been shown to be correlated with platelet count, which is a negative prognostic indicator in sepsis patients^10^.

One of the primary sources of extracellular histones during sepsis is are neutrophils during the formation of neutrophil extracellular traps (NETs), where a neutrophil releases intracellular material – namely DNA and associated histones^4,7^. Extracellular DNA and histones have been shown to be both proinflammatory and thrombogenic^10^. Circulating histones can result in activation of nearby platelets and leukocytes, can activate thrombin to exacerbate intravascular clot formation, and can initiate endothelial and epithelial cell death via Toll-like receptor-2 and -4 (TLR2 and TLR4) signaling^4,7,11,12^. Both Platelets and megakaryocytes have been shown to express TLR2 and TLR4^13^. Platelets have been shown to play a major role in leukocyte recruitment and NET-induction during infection and inflammation as well as NET propagation, as activated platelets stimulate NET formation and NETs also activate platelets. Substantial research has elucidated that platelets stimulate NET formation via both soluble and adhesive proteins in response to pathogenic stimuli (lipopolysaccharide and thrombin)^14–16^. While there are many mechanisms by which platelets are activated, dictating receptor expression and binding, platelet TLR4 has been shown to activate NET formation in response to bacteria in septic blood. Platelet TLR2/6 and 1/2 also triggers platelet activation, increased expression of CD62P (P-selectin) and stimulated platelet-neutrophil binding. Although this platelet-neutrophil interaction is meant to respond to pathogens, this also results in the initiation of coagulation via thrombin activation^17,18^.

In this paper, we explore whether MKs can be a source of circulating histones through the release of histone-rich platelets. We further explore whether platelets from patients with sepsis have significantly higher levels of platelet-associated histones (PAHs)^19^.

## Results

### Megakaryocytes contain extranuclear histones

**Figure 1:**
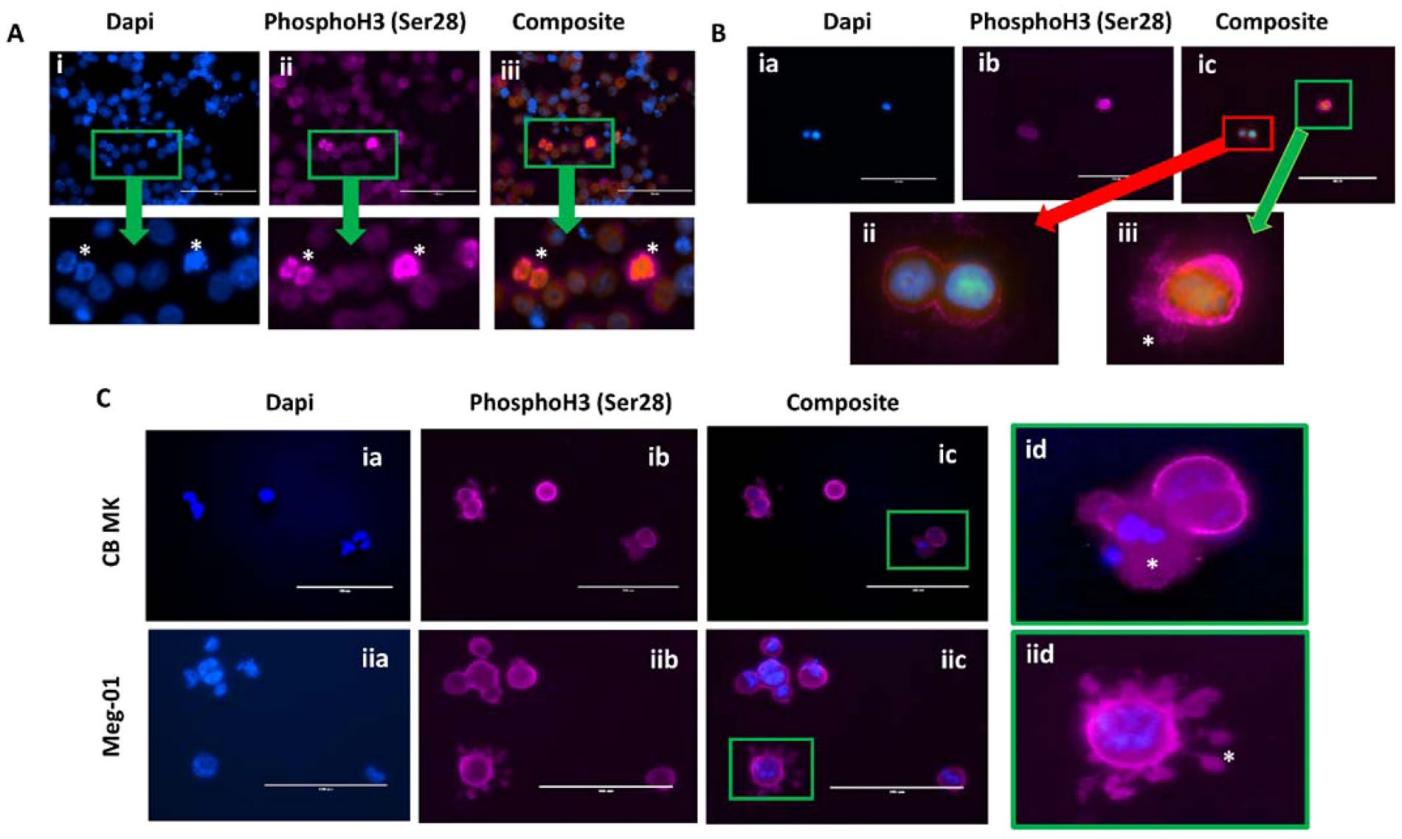
Megakaryocytes have extranuclear histones. Immunofluorescent images of megakaryocytes demonstrating extranuclear localization of PhosphoH3 (Ser28) histones. (**A**) Permeabilized Meg-01 cells are observed to have histone staining throughout the cell, including strong nuclear staining (specifically in cells with mitotic figures) (*). (**B**) CB MKs at day 10 of differentiation were fixed and permeabilized prior to staining and imaging. Cells on the bottom left panel (ii) appear to be quiescent and round with histone staining in the nucleus as well as around the cell membrane, while the cell on the bottom right panel (iii) has much stronger histone staining, particularly around the cell membrane and what appears to be pseudopods or proplatelet buds (*). (**C**) CB MKs (i) and Meg-01 cells (ii) were co-incubated for 1 hour with 3 μg/mL and 30 pg/mL *E. coli* LPS, respectively. The cells were then fixed, permeabilized, stained and imaged. The top right panel (id) is representative of cells that were observed to have a break in the cell membrane and release extracellular DNA and histones (*) in response to 3 μg/mL LPS. The bottom right panel (iid) appears to be a cell extending histone-positive pro-platelet buds (*) in response to 30 pg/mL LPS.

Immunofluorescent imaging used to localize histones within megakaryocytes showed that extranuclear histones are common in both Meg-01 cells and in cord blood-derived (CB) MKs (Fig. 1). Histone 3 phosphorylated at position serine 28 (PhosphoH3; Ser28; H3s28ph) was used as a histone marker. Ser28 is known to increase in response to activation of the MAPK pathway and during mitosis^20^. Light granular staining was seen diffusely throughout the Meg-01 cell membrane and cytoplasm, with an increase in staining at the nucleus of cells with mitotic figures (Fig. 1A). CB MKs were also positive for Ser28, with small, round quiescent cells having mainly cell membrane and cytoplasmic staining, while more activated cells appeared to have positive staining within cellular extensions at, what appears to be, the uropod of the cell (Fig. 1B). Upon stimulation with *E. coli* LPS, CB MKs were observed to form extracellular webs of histones and DNA; Meg-01 cells displayed histone staining within what appeared to be proplatelet buds (Fig. 1C).

BacMam technology was used to transfect Meg-01 cells with GFP-Histone 2B (H2B) to allow for a non-antibody-dependent method of confirming the presence of extranuclear histones within MKs. In most of the cells, H2B appeared to be primarily located within the nucleus. Rarely, extranuclear histone were visualized within the cytoplasm of the cell or within platelet-like particles within the cell culture (Supp. Fig. 1). The visualization of GFP-H2B is less likely to be artifact or background, as compared to antibody-dependent methods, such as immunofluorescent staining, because the GFP-H2B must be synthesized by the cell itself. It was observed that the cells which appeared to have cytoplasmic GFP-H2B concurrently had very strong GFP signal within the nucleus, which may either indicate that the cell is overexpressing the histone, or the cell is polyploid and approaching its apoptotic stage, where histones are likely to end up within the cytoplasm of the cell^2,21,22^. These results strongly suggest that both Meg-01 cells and CB MKs appear to have extranuclear histones, although perhaps H2B is not the most prominent extranuclear histone in these cells.

### Platelets contain histones

**Figure 2:**
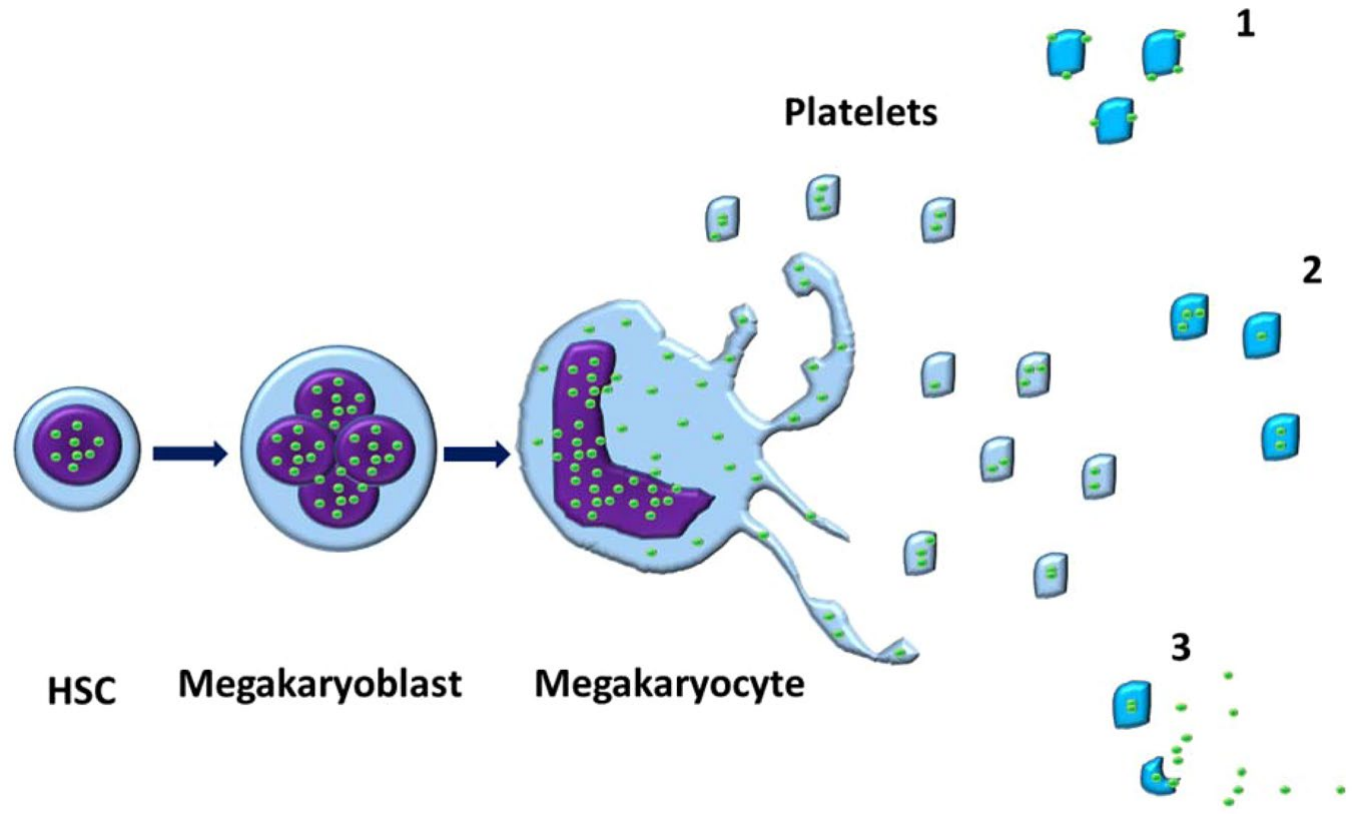
Megakaryocytes contain extranuclear histones, which are passed on to platelets. The proposed mechanism for the development of platelet-associated histones (PAHs). A mononuclear hematopoietic stem cell (HSC) differentiates into a megakaryoblast and undergoes endomitoses to form multiple nuclei. The nuclei (purple) contain various intranuclear histones (green circles). Once differentiation is complete and a mature megakaryocyte is made, the cell begins undergoing a controlled apoptosis, where the nuclear membrane develops pores and histones begin to leak into the cytoplasmic compartment. Upon the formation of proplatelet buds, the cytoplasmic and cell membrane-associated histones are also packed into the resultant platelets. The histones can be present in histones either primarily bound to the cell membrane (1), primarily within the cytoplasm (2), or a combination of the 1 and 2, where platelet activation results in the increased expression of histones on the cell membrane and possible release into the environment accompanied by degranulation.

**Figure 3:**
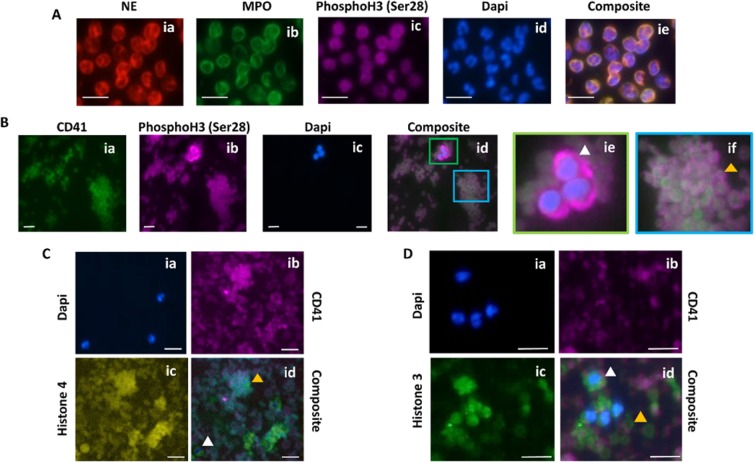
Immunofluorescent images of platelet-associated histones. Immunofluorescent images of white blood cells and platelets confirm the presence of platelet-associated histones. (**A**) Isolated human neutrophils were used as a positive control to demonstrate the specificity of the PhosphoH3 (Ser28) antibody to the nucleus of the cell. (**B**) Platelet-rich plasma was fixed, permeabilized, and probed for the presence of Ser28 and showed positive staining both within white blood cells (ie) and platelets (if). Notably, it appeared that the CD41 (green) platelet staining was towards the center of the platelets, while the Ser28 staining (pink) was concentrated around the periphery. (**C**) Platelet pellets were further probed with anti-Histone 3 (**C**) and anti-Histone 4 (**D**) antibodies. Platelets were noted to stain positive for both histone stains to various degrees. The occasional white blood cell that was present in the concentrated platelet pellet served as an internal stain positive control, confirming nuclear staining of histones 3 and 4. White blood cell, white arrowhead; Platelets, yellow arrowhead.

**Figure 4:**
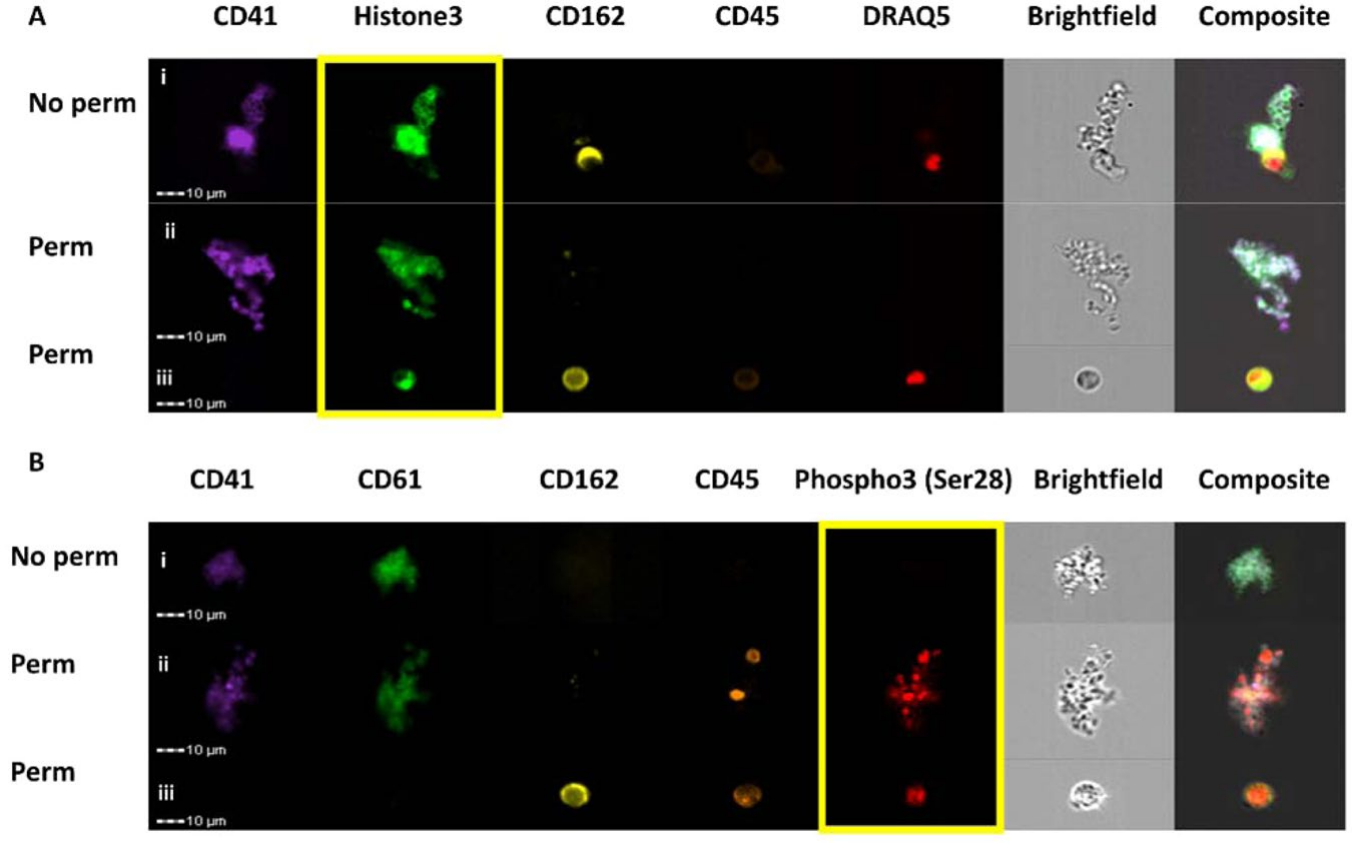
Imaging flow cytometry shows platelet-associated histones. Flow cytometry was used to probe for histones in both non-permeabilized (No perm) and permeabilized (Perm) platelets. CD41 and CD61 are used as platelet markers. CD162 and CD45 are used as leukocyte markers. Draq5 is used as a DNA marker to identify nucleated cells. In this figure, platelet clusters are shown. (**A**) Histone 3 appeared to be present around the platelet membrane in the non-permeabilized cells (i) and throughout the cell in the permeabilized cells (ii). A white blood cell used as an internal control has histone 3 nuclear staining (iii). (**B**) Phospho3 (Ser28) did not stain the non-permeabilized cells (i), but did stain the permeabilized cells (ii). A permeabilized white blood cell did stain positive for nuclear Phospho3 (Ser28) (iii). Yellow box is around the histone-stain channel.

Once the presence of extranuclear histones in MKs was demonstrated, we sought out to explore whether platelets also contain extranuclear histones (Fig. 2). Immunofluorescent imaging was used to probe whether peripheral blood platelets are positive for histones. Isolated neutrophils from healthy controls were used as a positive control for the Ser28 staining and confirmed the nuclear localization of this stain (Fig. 3A). Buffy coats were then stained with Ser28 and showed that platelets did appear to stain positive for histones; where Ser28 appeared to be primarily located to the platelet membrane, CD41 positively identified the platelet and showed diffuse staining throughout the platelet membrane and cytoplasm (Fig. 3B). Leukocytes in the buffy coat were used as an additional internal positive control for histone staining, demonstrating nuclear localization of the Ser28 marker. Platelets were then probed with a generic Histone 3 (H3) and Histone (H4) antibody to further confirm the presence of platelet histones (Fig. 3C,D). Imaging flow cytometry was then used to further test for the presence of platelet-associated histones (PAHs) in both permeabilized and non-permeabilized cells. Interestingly, platelets stained with the generic H3 antibody were positive in the non-permeabilized cells and appeared to be localized to the platelet membrane; whereas the permeabilized cells had staining throughout the entire platelet (Fig. 4A). While Fig. 4 demonstrates platelet clusters, single platelets were also noted to have histone staining as well. Ser28, on the other hand, did not stain the non-permeabilized platelets, but did appear to stain the permeabilized platelets (Fig. 4B), showing that different subtypes of histones may have different cellular localization within the platelet. These results suggest that Ser28, H3, and H4 are all PAHs.

**Figure 5:**
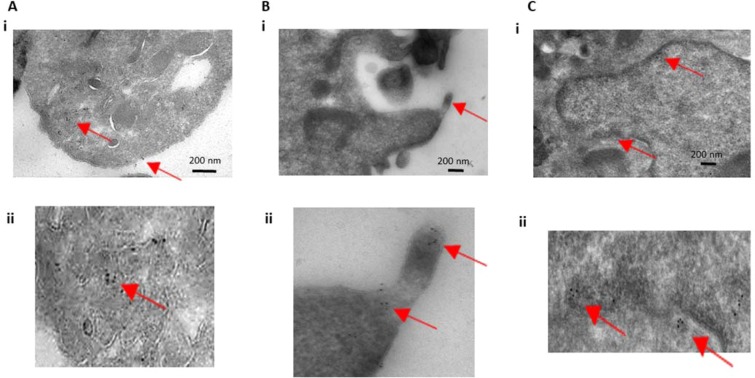
Transmission electron microscopy and immuno-gold labeling of platelet histones. Cells were immuno-gold labeled with anti-histone 4 antibodies and then imaged with transmission electron microscopy. (**A**,**B**) Platelets are shown to label positive for histone 4 in their cytoplasm as well as on their cell membrane. Magnified images are shown in the bottom panels (Aii and Bii). (**C**) HT-29 cells were also immuno-labeled and imaged as a positive control and showed nuclear labeling of histone 4. Magnification of the nuclear envelope with labeling is shown on the bottom panel (Cii). Red arrows point towards immuno-gold particles (6 nm).

Transmission electron microscopy (TEM) immuno-gold imaging was then used to attempt to determine the cellular localization of histones within platelets. Platelets were positive for H4 immuno-gold labeling (Fig. 5). Interestingly, the gold particles appeared to be diffuse throughout the cytoplasm of the cell, with pockets of more concentrated labeling, which tended to be either on the platelet membrane or on electron-dense areas of the TEM image. There was little-to-no immuno-gold labeling within the platelet granules or mitochondria. These findings suggest H4 is diffuse within the platelet cytoplasm and is does not appear to be packaged within platelet granules. The term “PAHs” is specifically used in the description of these findings because, although histones appear to be within the cytoplasm of the platelet, it is known that platelets can internalize proteins and particles and we cannot state that these histones originate from the platelet itself. Additionally, different histones may be localized to different compartments of the platelet and may interact with the environment by internal-external signaling, cell surface signaling, or extracellular release (Fig. 2).

### Histone quantification

PAH quantification was performed utilizing a combination of methods, including: absorbance and bead-based ELISA assays. The absorbance method for total protein and histone quantification results are shown in Supp. Table 1. The total protein was calculated with the following equation (Eq. 1):

1 $$(OD\,{0.42})/({1}\,mg/mL)=(measured\,OD\,at\,{230}\,nm)/(X\,mg/mL)$$

*X* = *total protein concentration*

Calf thymus histones were used as a positive control for this methodology and showed a relatively good agreement between the expected total protein concentration (10 mg/mL) and the measured total protein concentration (10.35 mg/mL). The Meg-01 histones were calculated to be at a concentration or 7.07 mg/mL and the two platelet extracts that were tested were calculated to be 0.18 mg/mL and 0.15 mg/mL. The samples were then evaluated for individual histone concentrations using their expected molecular weights and extinction coefficients (Supp. Table 2). The total histone concentration (H2A, H2B, H3, and H4) of the calf thymus, Meg-01 cells, and both platelet extracts were 9.75 mg/mL, 6.28 mg/mL, 1.51 mg/mL, and 1.17 mg/mL, respectively. Although the histone concentrations are close to the total protein concentrations for the calf thymus and the Meg-01 cells, they were about ten times higher than the protein concentrations for the platelet extracts. The lower detection limit of the absorbance reader was 0.04 mg/mL; therefore, we do believe that the concentration should be within the detectable range for the reader.

Next, we used a bead-based ELISA assay for the quantitation of histone 3 and a few specific histone 3 post-translational modifications (PTMs). (Supp. Table 3). Sequential dilutions of the Meg-01 purified histones were performed to verify the assay protocol as well as derive the histone concentration in the platelet extract sample (Supp. Fig. 2A & Supp. Table 4). The dilution curve for the Meg-01 cells fit a logarithmic line (Eq. 2) with an R-squared value of 0.99.

2 $$Y={614.9}\,\mathrm{ln}(X)+{462.9}$$

*Y* = *MFI, mean fluorescent intensity; X* = *histone concentration (ng/uL)*

Using Eq. 2, the MFI for the platelet histones was used to back-calculate the total histone concentration in the sample. This concentration was then used to calculate the total amount of H3 per platelet (Eq. 3), using an estimated input platelet count of 300 × 10^6^ platelets.

3 $$Y=X/A$$

*Y* = *ng/platelet; X* = *total His3 concentration (ng); A* = *platelet count (x10*^*6*^*)*

According to Eq. 3, the estimated His3 concentration in platelets was 0.42–0.46 ng/10^6^ plts. This protein concentration is within the range of other platelet protein concentrations, such as thrombospondin-1 (TSP-1) and platelet factor 4 (PF4) (31 + 12 ng/10^6^ plts and 12 + 5 ng/10^6^ plts, respectively)^23^. Total estimated concentration for each of the His3 modifications measured with the bead-based assay are listed in Supp. Table 4. Although we were able to calculate the best-fit line for total H3, this was not able to be done for the H3 PTSMs tested because we did not know the initial input of each of these PTSMs; therefore, these specific histones are expressed in terms of percent of total H3 expressed. As seen in Supp. Table 5, H3kme4 and H3k27me3 were the PTSMs expressed in the highest abundance in the Meg-01 cells, as compared to H3k56ac and H3s10ph. In the platelet extracts, the histone PTSM expression appeared to follow a similar trend as the Meg-01 cells, with both H3k56ac and H3s10ph appearing to be below the limit of detection (Supp. Fig. 2B). Here we have demonstrated that, in addition to total H3, we can also detect specific PTSMs within platelets. At this point in time, we cannot draw any conclusions as to what these specific PTSM patterns signify, as the histones tested were ‘total’ histone extracts and were not specific for any gene sequence.

### Sepsis results in increased platelet-associated histones (PAHs)

**Figure 6:**
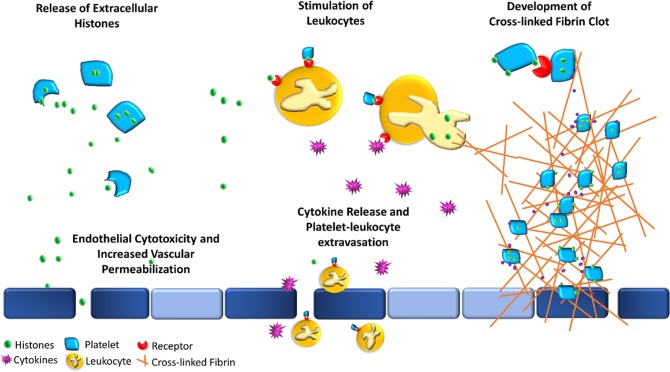
Proposed potential sequela of platelet-associated histones. Visual representation of some of the potential interactions of platelet-associated histones (PAHs) with the immune and coagulation system. Platelets (light blue polygons) may either release extracellular histones (green circles) upon stimulation or express them on their cell membranes. The release of extracellular histones (top left) may result in direct cytotoxicity of endothelial and epithelial cells (dark blue rectangles), resulting in both, increased vascular permeability, and a prothrombotic surface. The presence of PAHs and extracellular histones may serve as an additional binding antigen between platelets and leukocytes (orange circle) (top middle) via various receptors (red), including TLRs, and may result in the development of circulating platelet-leukocyte aggregates (PLAs) and stimulate the cells to increase production and release inflammatory cytokines (purple stars). These PLAs can then either diapedese across the endothelium or escape through the increase endothelial permeability into extravascular tissue (bottom middle) and further propagate inflammation. Thrombosis may be initiated and propagated through various mechanisms (right), where PAHs can bind to each other through TLR receptors (top right) which can result in subsequent platelet activation and an increase in thrombin generation and release (purple circles). Circulating histones are also themselves, prothrombotic and anti-fibrinolytic, promoting thrombosis. These PAHs and free circulating histones may also stimulate leukocytes, such as neutrophils, to form extracellular traps, further propagating inflammation and coagulation.

The presence of PAHs may play a significant role in the pathophysiology of sepsis through a variety of mechanisms, including extracellular histone release and resultant cytotoxicity and increase vascular permeability, increased platelet-leukocyte aggregate formation, and increased thrombogenicity (Fig. 6). To evaluate whether platelet-associated histones were increased in patients with sepsis, we performed imaging flow cytometry on isolated platelets. The markers that were evaluated included: CD34, which can either be increased if budding off of immature megakaryoblasts (i.e. immature platelet fraction) or can be decreased if platelets are activated, Draq5^+^ which may be present in some platelets if platelet-formation results in DNA-containing platelets, His3, which we hypothesize to be increase in patients with sepsis, and Draq5^+^His3^+^ cells, which may indicate that His3 and DNA are bound together within the platelet. Platelets were positively identified by gating for small CD41^+^ (GPIIb) cells. Platelet analysis was also further subdivided into ‘single’ platelets and ‘clustered’ or ‘aggregated’ platelets. The reasoning for this classification is because it is much more likely for platelets that are in aggregates to express higher levels of activation markers, which may or may not be related to the amounts of DNA or His3 expressed.

To account for the sample storage and preparation conditions of the patient plts, which included plt isolation followed by storage at −80 °C, we first evaluated the effect of these conditions on control plts from healthy patients, as we suspected some cell membrane rupture upon freeze-thawing (Supp. Figa. 3 & 4). When comparing the fresh platelets to the freeze-thawed platelets, there were changes in cell surface marker expression, for both single and clustered platelets, including: an increase in His3^+^ plts (1.8 + 0.6 and 28.9 + 1.7% His3^+^ plts vs. and 7.0 + 1.6 and 52.9 + 5.5% His3^+^ plts, respectively), an increase in CD34^+^ plts (2.0 + 1.8 and 28.9 + 1.7% CD34^+^ plts vs. and 9.4 + 1.9 and 52.9 + 5.5% CD34^+^ plts, respectively), a decrease in Draq5^+^ plts (4.9 + 6.0 and 47.4 + 17.7% Draq5^+^ plts vs. and 0.05 + 0.02 and 14.3 + 1.4% Draq5^+^ plts, respectively) and a decrease in Draq5^+^His3^+^ plts (0.5 + 0.9 and 25.3 + 4.6% Draq5^+^His3^+^ plts vs. and 0.02 + 0.01 and 13.9 + 1.3% Draq5^+^His3^+^ plts, respectively. There was also decrease in the percent of platelets that are within clusters or aggregates (51.1 + 17.8 vs. 24.1 + 3.7%, respectively). All these changes were significant with a p < 0.05. The same changes were seen in the single and clustered groups, although to a greater extent in the clustered, likely due to increased activation. At this point in time, we are not sure why CD34^+^ increased with freeze thawing, as it has previously been shown to decrease with platelet activation; this may be due to rupture of the platelet membrane upon thawing and increased exposure of internal CD34 resulting in increased CD34^+^ cells. Due to the differences in platelet phenotype with freeze-thawing, we treated all controls to freeze-thawing and used those values as the negative control for the sepsis patient evaluation; this would allow us to compare the relative changes between groups.

**Figure 7:**
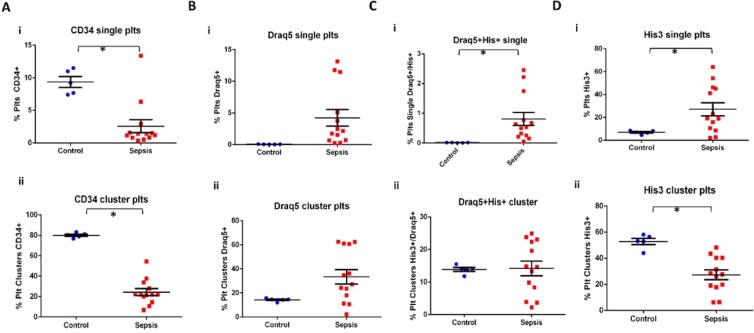
Flow cytometry analysis of platelets from patients diagnosed with sepsis. Flow cytometry was performed to explore the platelet phenotype of (**A**–**D**) Scatter plots with mean and standard error bars comparing fresh compared to freeze-thawed platelets for both single platelets (i) and clustered platelets (ii). Student unpaired t-test was performed, with significance defined as p < 0.05 according to a paired student t-test for all samples.

As expected, patients with sepsis did have a different platelet phenotype than the healthy controls (Fig. 7 and Supp. Figs. 5 & 6A). Three patients were not included in the statistical analysis: Patient 2 was not included in this analysis because, although this patient was diagnosed with sepsis, by the time the blood sample was collected, the urine culture was negative, and they were presumed to no longer have an active bacterial infection, Patient 13 did not have any positive bacterial culture and Patient 11 had a chronic septic condition and was on palliative care for longer than 30 days (Supp. Table 6). There were significantly more His3^+^ plts in the sepsis group as compared to the control group for the single plts (27.1 + 20.6 and 7.0 + 1.6% His3^+^ plts, respectively), whereas there were significantly less His3^+^ plts the clustered platelets (27.3 + 13.4 and 52.9 + 5.5% His3^+^ plts, respectively). There was no significant difference in Draq5^+^ platelets in the sepsis group as compared to the control group, for both single and clustered platelets (4.2 + 4.7 and 0.05 + 0.02% Draq5^+^ plts vs. 33.5 + 21.7 and 14.3 + 1.4% Draq5^+^ plts, respectively), although there appeared to be an upwards trend. CD34^+^ platelets were significantly decreased in patients with sepsis as compared to controls, for both single and clustered platelets (2.6 + 3.6 and 9.4 + 1.8% CD34^+^ plts vs. 24.4 + 12.3 and 80.1 + 2.4% His3^+^ plts, respectively). Draq5^+^His3^+^ platelets were significantly increased in the single platelets (0.8 + 0.8 and 0.02 + 0.01% Draq5^+^His3^+^ plts, respectively), but not in the clustered platelets (14.2 + 8.2 and 13.9 + 1.3% Draq5^+^His3^+^ plts). Notably, there was no significant difference in the number of platelet clusters between control and sepsis patients (21.1 + 11.5 and 24.1 + 3.7% clustered plts, respectively), further supporting that the changes in the platelet phenotype are not simply due to increase platelet activation and aggregation. Interestingly, His3^+^ platelets in sepsis patients also appeared to have two different populations, a His3^lo^ and a His3^hi^ population; these two populations were not seen in the control groups (Supp. Fig. 5F).

**Figure 8:**
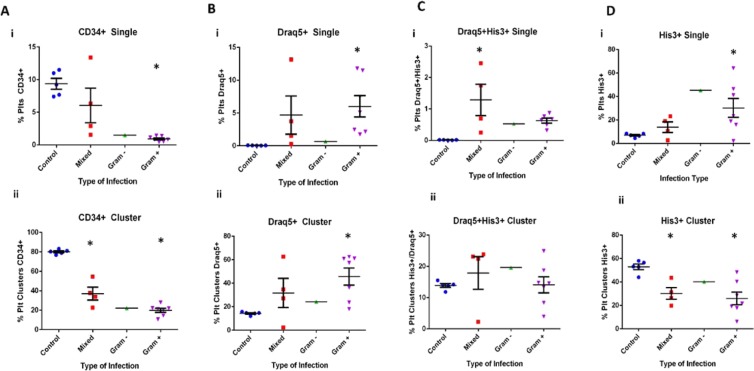
Platelet phenotype is associated with type of infection. Flow cytometry was performed to evaluate platelet phenotype from patients diagnosed with sepsis. (**A**–**D**) Platelet phenotype was also compared for patients with different types of infection: Gram positive (Gram +), Gram negative (Gram −), or Mixed. Single platelets (i) and clustered platelets (ii) were evaluated. One-way ANOVA testing was performed comparing the type of infection to the control group, with significance being defined as p < 0.05 (*). Statistics were not performed on the Gram – group as there was only one patient in this group.

Changes in platelet phenotype were also explored over time in patients with sepsis (Fig. 8 and Supp. Fig. 6B,C and Supp. Table 6). Nine patients were followed on sequential days during hospitalization and it was noted that His3^+^ platelets were significantly higher during early sepsis than on subsequent days in six of the patients (Supp. Fig. 6C). There were two patients that did not follow this trend: patients 11 and 12, which were both diagnosed with unresolved, intermittent sepsis symptoms requiring repeated hospitalization, lasting weeks-months, and, prior to sample collection, were under ‘palliative care’ and did not recover from this event. Platelet phenotype was also evaluated with respect to the type of infection: Gram positive, Gram negative and mixed infections (Gram positive and Gram negative bacteria) (Fig. 8 and Supp. Fig. 6B-). His3^+^ cells appeared to be at higher levels in patients diagnosed with Gram positive bacterial infections, as compared to gram negative infections (Fig. 8D). Cumulatively, these results suggest that, in addition to increased platelet activation, as noted by decreases in CD34^+^ platelets, there was an increased number of platelets that had associated DNA (Draq5) and His3. Interestingly, the percentage of CD34^+^ and His3^+^ did not correlate with platelet count or white blood cell count in patients with sepsis (Supp. Figs. 7 & 8); this suggests that the presence of PAH and platelet activation may be independent predictors of sepsis.

## Discussion

Histones are an essential building block for nuclear material, helping form chromatin and acting as control switches for the transcription of DNA. In addition to their essential role in transcriptional regulation, histones also have important roles outside of the nucleus, including serving as damage-associated molecular patterns (DAMPs)^4,5^. When histones are outside of the cell, they bind to several different receptors on white blood cells and platelets, such as toll-like receptor 2 and 4 (TLR2 and TLR4)^7,24–26^. Extracellular histones are typically degraded by a variety of enzymes, such as histone deacetylases (HDACs); but when they are significantly increased they can bind and activate the surrounding cells and may result in white blood cell activation, platelet activation, and endothelial cell damage^27^. Circulating histones can be significantly increased during sepsis and are thought to play an integral role in the pathophysiology of severe systemic inflammation and immunothrombosis^6,27–30^. Experimental treatment with TLR4 receptor inhibitors and HDACs in animal models of sepsis have helped support this mechanistic hypothesis^31–36^. Up until now, platelets have been shown to bind extracellular histones through TLR2 and TLR4 receptors and liberate histones from bound DNA through the release of highly positively charged platelet factor 4 (PF4), but platelets have not been explored as a potential source of circulating histones themselves^26,37^. In this paper, we demonstrated that megakaryocytes (MKs) commonly contain extranuclear histones, and that these ‘parent’ MKs may transmit these histones to their ‘daughter’ platelets, resulting in an additional source of circulating histones during severe inflammation and platelet activation, such as in sepsis. We also demonstrated that patients with sepsis appear to have increased platelet-associated histones (PAHs)^19^.

Although histones are most commonly located within the nucleus of a cell, extranuclear histones have been demonstrated in a variety of cells and in several conditions. The role histones outside of the nucleus is not completely clear, but they have been shown to be extremely proinflammatory and even prothrombotic^2–5^. There are a number of mechanisms by which histones can be found outside of the nucleus, including extracellular trap formation, apoptosis, via escape or leakage through nuclear membrane pores, and as a functional protein within the mitochondrial membrane^2,21,22,38^. As hematopoietic stem cells (HSCs) differentiate into MKs and mature into platelet-producing cells, they typically undergo controlled apoptosis^39–45^. During this, typically, caspase-mediated apoptosis, it is not surprising that MKs likely develop nuclear membrane pores, where intranuclear material, such as histones, may leak out into the cytoplasm^21,22,46^. In this paper, we have demonstrated that MKs do in fact commonly contain histones in both their cytoplasm and along their cell membrane. The exact role of the histones in these compartments, if any, is not currently understood.

Platelets are organized buds composed of MK cell membrane and cytoplasmic material; therefore, it is not out of the question for extranuclear histones within the cell membrane and cytoplasm of the MK to be a part of the platelet progeny (Fig. 2). Platelets have been shown to bind to extracellular histones via TLR receptors, potentially playing a role in the proinflammatory and prothrombotic nature of extracellular histones during sepsis and its’ related complications^4,16,26^. Up until now, platelets have not been explored as potential sources of histones themselves. One paper by Stewart D.I.H. *et al*.^47^, did show that protein isolation of platelet pellets contained small concentrations of histone proteins, but at the time this was attributed to likely contamination of the platelet pellet with nucleated white blood cells and was disregarded^47^. Although we were able to confirm these findings through histone purification and quantitation, there is always the possibility that there were contaminating nucleated cells within our platelet pellets as well. More suggestive proof of platelet-associated histones was demonstrated by electron microscopy imaging showing that platelets stain positive for H4 in both the cytoplasm and cell membrane.

The quantity of histones that are present within the platelet compartment and cell membrane appear to be on the same scale as other platelet proteins, such as thrombospondin-1 and platelet factor 4, suggesting that circulating histones from platelets may be detectable as circulating biomarkers and contribute to the pool of extracellular histones. Although platelets may be capable of contributing to the extracellular histone pool, because they are anucleate cells, in conditions such as sepsis where neutrophils release histone-rich extracellular traps (NETs), platelets are likely not the primary source of circulating histones. Additionally, recent literature has demonstrated that specific histones have different effects, such as the pro-inflammatory and pro-thrombotic effect of citrullinated H3 released by NETs and the cytotoxicity and platelet activation of H4^11,48^. Therefore, to truly understand the effect of MK- and platelet-derived histones, the various histone combinations and associated post-translational modifications (PTSMs) need to be tested for related effects on the immune and coagulation system. Even more interesting is the possibility of platelet histone phenotype changing based on the stimulation and method of platelet generation from the parent megakaryocyte, as there are many mechanisms for platelet-genesis that may result in different histone and PTSM packaging within the platelets^49^.

We next explored whether there was an association between the presence of PAHs and sepsis. Our results demonstrated that there was an increase in His3^+^ platelets, as well as an increase in Draq5^+^ platelets and Draq5^+^/His3^+^ platelets. Although this is suggestive that there may be an increase in PAHs and DNA content during sepsis, we cannot rule out that the histones and platelets were not just bound to the platelet membrane from the circulation, as plasma histones and DNA are known to be increased during sepsis and platelets are known to be able to internalize proteins and bind histones^4,11^. There was also a significant decrease in CD34^+^ platelets, which is suggestive of platelet activation, although platelet clusters, another sign of platelet activation, was not increased in sepsis patients^50^. Interestingly, this unique phenotype lacking platelet aggregation but having an increase in platelet activation is consistent with previous research demonstrating that platelets from patients with sepsis are more likely to spontaneously activate but are less likely to aggregate in response to various platelet agonists^51–54^. This platelet phenotype was also observed to revert to ‘normal’ over time as a patient was treated for sepsis, suggesting that this may also be an early indicator of sepsis. The determination of the exact stage of sepsis at which the platelet phenotype begins to change would require a more thorough study, including pre- and post- sepsis sample analysis. The increase in PAHs may also be another mechanism by which platelets form platelet-leukocyte aggregates (PLAs) and are increasingly cleared or sequestered, leading to peripheral thrombocytopenia; both of which are known consequences of sepsis^46,55–57^.

In conclusion, this set of experiments has demonstrated that platelets may contain extranuclear histones, and that PAHs are increased in patients with sepsis^19^. Due to the small concentration of platelet histones that were measured (ng/10^6^ plts), platelets are likely not a primary source of plasma histones during sepsis; although, when platelets are activated, whether the histones are released, or they are brought to the plasma membrane, the PAHs may be stimulatory enough to propagate intravascular inflammation and thrombosis as demonstrated in the proposed potential interaction of PAHs with the inflammatory and coagulation system in Fig. 6. In this study, it was not possible to determine whether the PAHs in sepsis patients originated from the parent MK or were bound from the plasma matrix and internalized, such as the internalization of neutrophil-derived extracellular histones. Future studies to further explore this potential source of circulating histones during sepsis includes testing PAHs for their proinflammatory and prothrombotic properties and identifying the original source of the histones. Additionally, because platelets appear to have various H3 PTSMs, exploring whether these are bound to DNA or whether the different histone PTSMs have specific interactions with the immune and coagulation system, as seen with CitH3, would also be very valuable.

## Methods and Materials

All information on cell culture, platelet and white blood cell isolation, immunofluorescence imaging, histone-2B BacMam transfection of Meg-01 cells, histone purification and quantification, flow cytometry, transmission electron microscopy and immune-gold labeling, patient sample collection, and statistical analysis is available in the SI Appendix. Discarded venous blood from patients diagnosed with sepsis was collected and evaluated for platelet-associated histones in accordance with the collection protocol approved by the Partners Institutional Review Board and the Massachusetts Institute of Technology Committee on the Use of Humans as Experimental Subjects (IRB protocol numbers, MGH No: 2014P002087; MIT No:150100681R001). Patient consent was not required for sample collection due to the use of discarded and de-identified blood samples. In brief, as blood was collected in EDTA tubes as part of normal clinical treatment, once the clinical laboratory testing was completed, an aliquot of this blood was used for experimental analysis. No blood draws and no extra blood tubes were collected to complete this study. All methods were carried out in accordance with relevant guidelines and regulations.

### Significance Statement

Histones are traditionally contained safely within the intranuclear compartment. The role of neutrophil-derived circulating histones in the propagation of inflammation and coagulation during sepsis is well known. In this study, we demonstrate that megakaryocytes (MKs) frequently contain extranuclear histones and that they may transfer these histones to their platelet progeny. Further, we show that platelet-associated histones (PAHs) are increased in patients with sepsis and appear to correlate with the type of infection present. These results suggest that MKs and platelets may contribute to circulating histones during sepsis.

## Supplementary information

Supplementary Information.
