# Supplementary material for: Megakaryocytes contain extranuclear histones and may be a source of platelet-associated histones during sepsis
Source: Sci Rep. 2020 Mar 12;10:4621. doi: 10.1038/s41598-020-61309-3 (PMC7067782; doi:10.1038/s41598-020-61309-3)
Supplement: Supplementary file 1 — Supplementary Information. [file 41598_2020_61309_MOESM1_ESM.pdf]

Supplementary Information

Megakaryocytes contain extranuclear histones and may be a source of platelet-associated histones during sepsis.

Galit H. Frydman[1,2*], Shannon Tessier[2], Keith H. K. Wong[2], Charles R. Vanderburg[3], James G. Fox[1], Mehmet Toner[2], Ronald G. Tompkins[2], Daniel Irimia[2,*]

[1]Division of Comparative Medicine and Department of Biological Engineering, Massachusetts Institute of Technology, Cambridge, Massachusetts, United States of America.

[2]BioMEMS Resource Center and Center for Surgery, Innovation & and Bioengineering, Department of Surgery, Massachusetts General Hospital, Boston, Massachusetts, United States of America.

[3]Harvard Neurodiscovery Center. Harvard Medical School. Boston, Massachusetts, United States of America.

Supplemental Methods and Materials
Supplemental Figures 1-8
Supplemental Tables 1-6

Methods and Materials

Cell culture

Two sources of megakaryocytes were used for these experiments: Cord blood-derived $CD34^+$ cells and a megakaryoblastic cell line (Meg-01). Cord blood $CD34^+$ stem cells were purchased and cultured in StemSpanII media with the megakaryocyte supplemental cytokines, according to the culture and differentiation protocols from Stemcell Technologies (Stemcell Technologies, Vancouver, BC, Canada). TheMeg-01 cells was purchased and cultured in RPMI with 10% FBS, according to the standard culture protocols from ATCC (American Type Culture Collection, Manassas, VA). All cells were incubated in sterile conditions at 37°C.

Platelet and white blood cell isolation

For the evaluation of blood cells for the presence of histones, blood was acquired from Research Blood Components, LLC (Boston, MA). As a positive control for histone staining, neutrophil isolation was performed. For this, blood was collected in ACD BD Vacutainer tubes (Becton Dickinson, Franklin Lakes, NJ, USA). Neutrophils were isolated from whole blood using a negative-selection protocol. In brief, neutrophils were isolated using a density gradient with HetaSep (Stemcell Technologies) and then purified with EasySep Human Neutrophil Kit (Stemcell Technologies), following the manufacturer's protocol. Neutrophil purity was assessed to be >98%, and cell count was performed using a hemocytometer. Neutrophils were subsequently resuspended in IMDM with 20% FBS (Thermo Fisher Scientific). . Platelets were prepared from blood collected into 2.9 ml 3.2% trisodium citrate Vacutainer tubes (Sarstedt, N¨umbrecht, Germany). Platelet-rich plasma (PRP) was first prepared by centrifugation of the

whole blood at 210 g, 22°C, for 20 min. The PRP supernatant was gently pipetted into a new tube and 20% volume ACD solution was added (Boston Bioproducts, Ashland, MA, USA) and then centrifuged at 1900 g, 22°C, for 10 min. The platelet-poor plasma (PPP) was removed, leaving a platelet pellet which was resuspended in Hepes-Tyrode buffer with 20% volume ACD solution (Boston BioProducts).

Immunofluorescence Imaging

Both Meg-01 and cord-blood derived MKs (CB MKs) were fixed and stained for the evaluation of cellular histone localization using immunofluorescence staining. Platelet pellets were prepared as described in the 'Blood cell sample preparation' section above. CB MKs, at day 14 of differentiation, and platelet pellets were fixed with 4% paraformaldehyde for 30 minutes and then concentrated onto a poly-lysine coated slide using the Cytospin 4 cytocentrifuge (Thermo Fisher Scientific), for 5 minutes at 1250 rpm, rinsed once with Deionized (DI) water and stored at -80°C until staining and evaluation. For the LPS-stimulation of Meg-01 cells, Meg-01 cells were co-incubated with various concentrations (30 pg/mL and 3 ug/mL) of *E. coli* lipopolysaccharide (LPS) (O111:B4; Sigma Aldrich, St Louis, MO), for 60 minutes at 37°C on poly-lysine coated slides (Sigma Aldrich, St Louis, MO, USA) and then fixed with 4% paraformaldehyde (Santa Cruz Biotechnology, Dallas, TX, USA). The slides were then rinsed with DI water and stored at -80°C until staining and evaluation. For staining, the slides were thawed at room temperature and blocked with 5% donkey serum (Jackson Immunoresearch) for 2 hours. The slides were rinsed three times with PBS and then treated with various combinations of the following primary antibodies for two hours at room temperature: rat anti-Histone H3 (phospho S28) antibody (HTA28; Abcam, Cambridge, MA, USA) at 1:500, rabbit anti-Histone

H4 (39270; Active Motif, Carlsbad, Ca, USA), rabbit anti-Histone H2A.X (Active Motif), rabbit anti-Histone 3 (ab1791; Abcam, Cambridge, MA, USA), and mouse anti-Histone 3 (96C10; Cell Signaling Technology, Danvers, MA, USA) at 1:200. The slides were then rinsed three times with PBS and incubated with various combinations of the following secondary antibodies: mouse anti-human CD41 (GPIIb) 488 or 647 (Biolegend, San Diego, Ca, USA) at 1:250, donkey anti-rabbit 488, donkey anti-rat 647, and donkey anti-mouse 568 (Life Technologies) at 1:500 for 30 minutes at room temperature. Slides were then rinsed three times with PBS and covered with Vectashield antifade mounting medium with DAPI (Vector Labs, Burlingame, Ca, USA). The cells were then imaged with one of two fluorescent microscopes: Life Technologies EVOS FL (Thermo Fisher Scientific) or Nikon Eclipse 90i microscope (Nikon Instruments Inc., Melville, NY).

Histone-2B BacMam transfection of Meg-01 cells

Meg-01 cells were transfected with GFP-Histone 2B (H2B) for localization of intracellular histone. Meg-01 cells were transfected with CellLight Histone 2B-GFP (BacMam 2.0, ThermoFisher Scientific) at 30 uL/mL for 48-72 hours at 37°C. Cell were then labeled with MitoSox Red mitochondrial superoxide indicator (ThermoFisher Scientific) at 1:1000 Hoechst at 1:2000 for 30 minutes. Cells were pelleted at 1,900 x g for 10 minutes and rinsed twice with PBS. Cells were then concentrated onto a poly-lysine coated slide using the Cytospin 4 cytocentrifuge (Thermo Fisher Scientific), for 5 minutes at 1250 rpm. Slides were rinsed with DI water, cover slipped, and imaged on an EVOS fluorescent microscope.

Histone purification

Histones were extracted from both isolated platelet pellets and Meg-01 cells for histone quantification. For sample preparation, venous whole blood was collected in acid citrate dextrose (ACD) tubes (Research Blood Components LLC, Boston, MA, USA). Platelet pellets were isolated as described in the section above, ‘Blood cell sample preparation’. Meg-01 cells were pelleted down at 1900 x g for 10 minutes. For histone purification the Active Motif Histone Purification Mini Kit (Active Motif, Carlsbad, CA) was utilized per the manufacturer’s instructions. Briefly, platelet and cell pellets were washed twice with hepes-tyrode buffer and 20% acid citrate dextrose buffer (Boston BioProducts, Boston, MA, USA). Pellets were then re-suspended in ice-cold Extraction Buffer. Samples were homogenized in the Extraction Buffer for 30 minutes and then left overnight at 4°C. The samples were then spun at maximum speed (18,000 x g) in a microfuge for 5 minutes at 4°C. The supernatant, containing the crude histones, was then transferred to a new tube and neutralized with 5X Neutralization Buffer to a pH of 8. One aliquot of crude histones was saved for downstream analysis at -80°C. Spin columns provided by the kit were then equilibrated and the crude histones along with the Elution Buffer were run through the columns at 500 x g for 3 minutes. One aliquot of the eluted histones was saved for downstream analysis at -80°C. Histones were then precipitated with perchloric acid. Briefly, histones were precipitated overnight at 4°C with 4% perchloric acid. The histones were then spun down at maximum speed for 1 hour at 4°C. The pellet was washed with 1 mL cold 4% perchloric acid, followed by two washes in ice cold acetone with 0.2% HCl, and then twice in ice cold acetone. The resulting pellet was air dried for 10 minutes and then re-suspended in sterile water. All histones and aliquots were stored at -80°C until the samples were analyzed.

Histone quantification

Histone quantification was performed using two methods: absorbance measurements and bead-based ELISAs. For the absorbance measurements, both total protein and histone-specific absorbances were performed as follows using the DS-11 Spectrophotometer (DeNovix Inc., Wilmington, DE, USA): 230 nm for total protein, 280 nm for histone quantification with the following molecular weight extinction coefficients, as recommended by Active Motif (**Supp. Table 1**). Histones purified from calf thymus (Sigma-Aldrich, St. Louis, MO, USA) were used as a positive control at 2 mg/mL. For histone quantification using ELISAs the Luminex Histone H3 PTM Multiplex Assay (Active Motif) was used according to the manufacturer's protocol. For sample preparation, serial dilutions of Meg-01 and platelet pellet purified histones were prepared. For the positive control, the purified histone 3 protein that was provided with the kit was used in this assay. Both total Histone 3 (H3) and H3 post-translational modifications (PTSMs) were evaluated for each sample (**Supp. Table 2**). Briefly, the samples were co-incubated with multiple magnetic beads, which were conjugated to antibodies targeted to specific H3 PTSMs, on a shaker plate for 2 hours. The beads were then washed 3 times, incubated with biotinylated anti-H3 antibodies on a shaker plate for 30 minutes, washed 3 times and then incubated with streptavidin-phycoerythrin for 30 minutes, followed by a final set of washes. Samples were read by using a Flexmap 3D (Luminex Corp., Austin, TX). The standard curve was generated by using 5-parametric-curve fitting with platform-specific software (xPONENT Software Solutions, Luminex). Samples were run in duplicate on each plate for quality control.

Transmission electron microscopy and immune-gold labeling

For transmission electron microscopy, cells were fixed in 2.5% gluteraldehyde, 3% paraformaldehyde with 5% sucrose in 0.1M sodium cacodylate buffer (pH 7.4), scraped, pelleted in a small eppendorf tube. Wash with 0.1M sodium cacodylate buffer 3x, post fixed in 1% OsO_4 in veronal-acetate buffer 1 hour. The pellet was stained overnight with 0.5% uranyl acetate in veronal-acetate buffer, dehydrated and embedded in Embed 812 resin. Sections were cut on a Leica Ultracut UCT microtome with a Diatome diamond knife at a thickness setting of 50 nm, stained with uranyl acetate, and lead citrate. The sections were examined using a FEI Tecnai spirit at 80KV.

For the immunogold labeling protocol, the protocol was as follows. (21) The cells were fix mildly Using PLP (paraformaldehyde / lysine / sodium periodate) fixative for 4 hours. Cells were pelleted and trimmed into small (<1mmsq) blocks. Infused with a cryo-protectant for at least one hour (PVP/ sucrose). Blocks were mounted onto cryo-pins, and snap frozen in liquid nitrogen. Ultrathin sections were cut at -110 degrees C with a Leica UC7 equipped with a FC7 cryo-stage using a glass knife, and immunolabeled, stained and embedded using the Tokuyasu technique. For the immmunolabeling, rabbit anti-histone 4 antibody (39270; Active Motif) was used. The material was examined using a FEI techni Spirit biotwin.[1]

Patient sample collection

Discarded venous blood from patients diagnosed with sepsis, according to their medical records, was collected and evaluated for platelet-associated histones in accordance with the collection protocol approved by the Institutional Review Board (IRB protocol numbers, MGH No: 2014P002087; MIT No:150100681R001). Patient consent was not required for sample collection

due to the use of discarded blood samples and all methods were carried out in accordance with relevant guidelines and regulations. In brief, as blood was collected in EDTA tubes as part of normal clinical treatment, once the clinical laboratory testing was completed, an aliquot of this blood was used for experimental analysis. No blood draws and no extra blood tubes were collected to complete this study. A patient was categorized as having sepsis when one of the diagnoses for the patient was 'sepsis' according to their medical record and when there was a confirmed infection via positive culture as part of their medical diagnosis. Twenty-one patient samples were evaluated with 16 sepsis-positive samples (age 36-85 yrs., 4 females, 12 males) collected from the Massachusetts General Hospital (Boston, MA) (**Supp. Table 6**) and 5 healthy control samples purchased from Research Blood Components (Research Blood Components, LLC, Boston, MA), age and sex not known. Platelet pellets were isolated as described in the 'Platelet isolation' section from venous blood collected in vacutainer EDTA tubes and then frozen at -80°C until analyzed. Flow cytometry was performed as described below, in the 'Flow cytometry' section. For the assessment of histone expression, platelet pellets were either treated with 4% paraformaldehyde fixation with 0.1% Triton™ X-100 or with 4% paraformaldehyde alone. The pellets were washed prior to staining and evaluating. To assess for the effect of freeze-thawing on the patient platelet pellets, one set of control samples (n=5) was divided into two aliquots and one was run "fresh" without freeze-thawing and the other was freeze-thawed. Both samples were pre-treated with 4% paraformaldehyde fixation with 0.1% Triton™ X-100 permeabilization. One set of samples was then frozen at -80°C. For the sepsis sample evaluation, platelet pellets were pre-treated with 4% paraformaldehyde fixation with 0.1% Triton™ X-100 permeabilization, pellets were stored at -80°C until ready to evaluate. They were then thawed and

washed prior to staining and evaluation. Samples were evaluated as described in the section below, 'Flow cytometry'.

Flow cytometry

Flow cytometry was performed to evaluate cells and platelets for histone expression. Cultured Meg-01 cells, isolated platelets, and whole blood were fixed with 4% paraformaldehyde with or without 0.1% Triton™ X-100 detergent (Sigma-Aldrich) for permeabilization for a total of 10 minutes. For the whole blood samples, platelets were isolated as described above in the 'Platelet Isolation' section prior to fixing. After fixing, samples were then pelleted down at 1,900 x g for 10 minutes and re-suspended in PBS. Cells were first incubated with primary, unconjugated antibodies, rabbit anti-Histone 3 (ab1791; Abcam) or rat anti-Histone H3 (phosphoS28) antibody (HTA28; Abcam) at 1:400 for 20 minutes and were then washed twice with PBS. Cells were then stained with a combination of conjugated primary antibodies and secondary antibodies for 15 minutes, including: mouse anti-human CD41 PacBlue, mouse anti-human CD45 594, mouse anti-human CD162 PE, mouse anti-human CD62P PE, mouse anti-human CD61 FITC (Biolegend, San Diego, Ca), and goat anti-rabbit 488 or donkey anti-rat 647 (Thermo Fisher Scientific, Waltham, MA) at 1:200 for 15 minutes, with the exception of CD41 which was at a concentration of 1:100 for 15 minutes. Cells were then stained with Draq5 (Thermo Fisher Scientific, Waltham, MA) at a concentration of 1:10000 for 5 minutes. Data was obtained through the Amnis ImageStreamX Mark II imaging flow cytometer and INSPIRE Software (EMD Millipore, Billerica, MA). The accompanying IDEAS Software was used to perform data analysis. Data is reported as the percent of the total cell population that stained positive for the specific marker.

Statistical analysis

Platelet were evaluated both as ‘singles’ and as ‘clusters’ to parse between the more activated platelets that are more likely to be present as clusters and individual platelets in circulation. Statistics were performed using both Excel (Microsoft, Redmond, WA, USA) and GraphPad Prism Software (GraphPad Software, Inc.). Either one-way ANOVA or student t-tests were performed to compare between conditions. Pearson’s correlation was used to determine whether there was a correlation present between platelet phenotype and platelet count or white blood cell count. A p-value of <0.05 was considered significant.

References:

1. Tokuyasu K.T. Immunochemistry on ultrathin frozen sections. *Histochem. J*. **12**, 381-403 (1980).

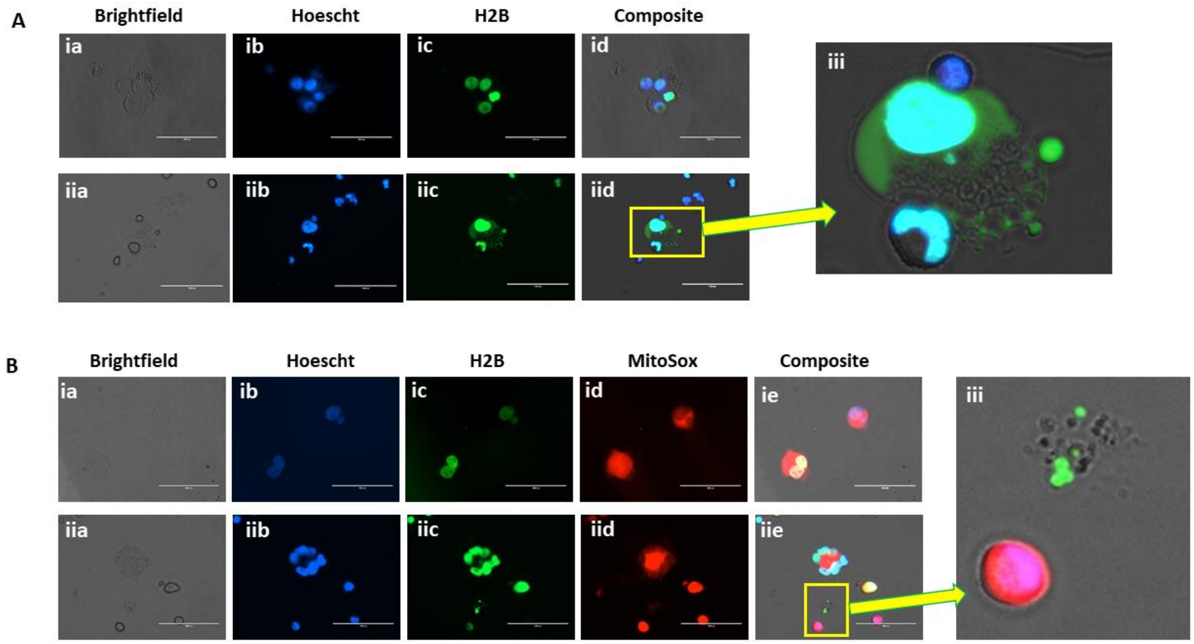

Supplemental Figure 1: GFP-H2B expressing Meg-01 cells have extranuclear histones. Meg-01 cells were transfected with GFP-Histone 2B (H2B) BacMam to explore the cellular localization of H2B in megakaryocytes. (A) H2B was mostly observed to be restricted to the nucleus within Meg-01 cells (i); although, occasionally cells were noted to have some extranuclear histones, such as the cells in the panel on the right (ii). (B) Cells were co-incubated with MitoSox red to show the cytoplasmic location of metabolically active mitochondria. While H2B was normally within the nucleus and within the boundaries of the cell membrane, isolated platelet-like particles with H2B expression were noted as well (ii).

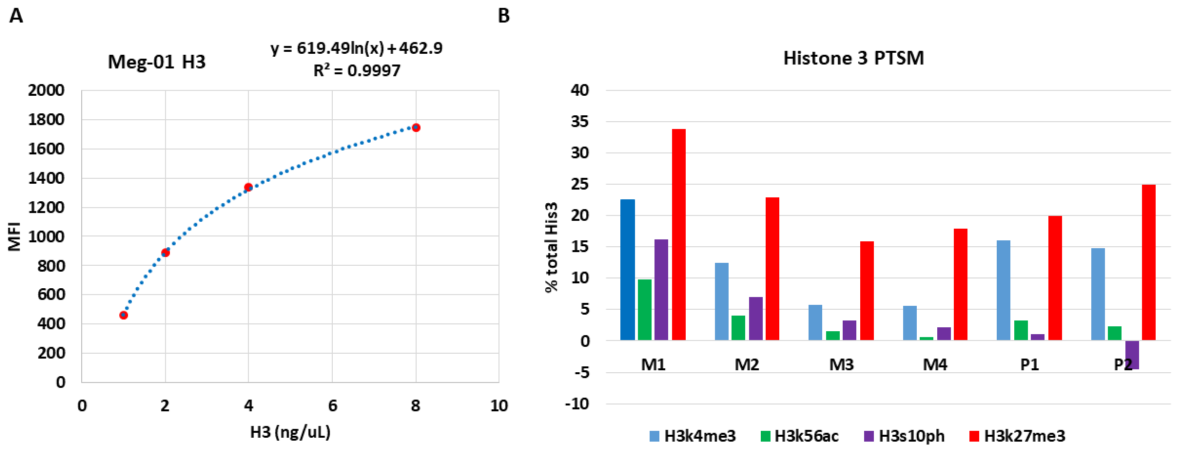

Supplementary Figure 2: Histone quantification using bead-based ELISA assays.

Histones extracted and purified from Meg-01 cells and platelet pellets were quantified. (A) Meg-01 cell histone-extract serial dilutions were run on the ELISA assay. His3 concentration based on absorbance measurements were used to extrapolate the histone concentration input (ng/uL) in each dilution. These concentrations were then plotted against the mean fluorescence intensity (MFI) reading from the ELISA assay. The points were then fit with a logarithmic best-fit line, which has an R^2 value of >0.99. (B) The Meg-01 and platelet histone extracts were also assessed for various H3 PTSMs. These are expressed in terms of % of total H3 based on MFI readings. M1, Meg-01 at a 1:250 dilution; M2, Meg-01 at a 1:500 dilution; M3, Meg-01 at a 1:1000 dilution; M4, Meg-01 at a 1:2000 dilution; P1, Platelet at a 1:25 dilution; P2, Platelet at a 1:50 dilution.

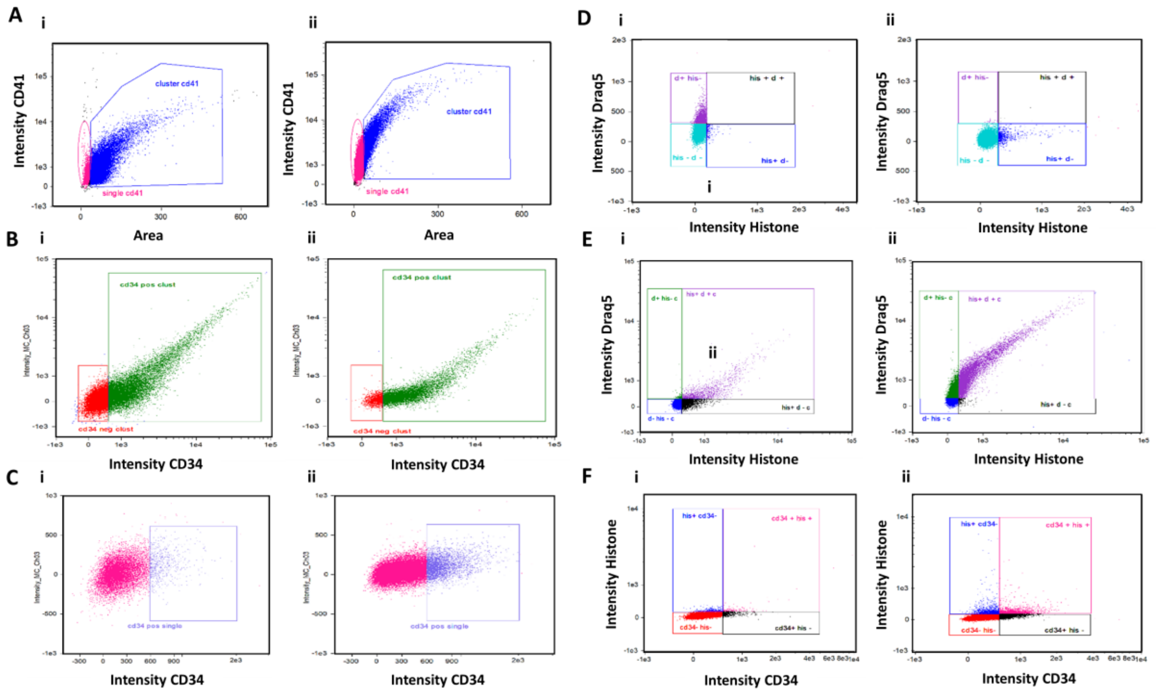

Supplementary Figure 3: Flow cytometry comparison of fresh versus freeze-thawed platelets.

Flow cytometry was performed on both fresh and freeze-thawed platelets, in order to control for the effects of storage conditions on the patient platelet analysis. (A-F) Representative scatter plots of flow cytometry results comparing fresh platelets (i) and freeze-thawed platelets (ii). (A) CD41 compared to area of cell identifies single versus clusters of CD41 platelets; (B) CD34 intensity in CD41 positive clusters; (C) CD34 intensity in Cd41 positive singlets; (D) Intensity of Draq5 and Histone in CD41 positive singlets; (E) Intensity of Draq5 and Histone in CD41 positive clusters; (F) Intensity of CD34 and Histone in CD41 positive singlets.

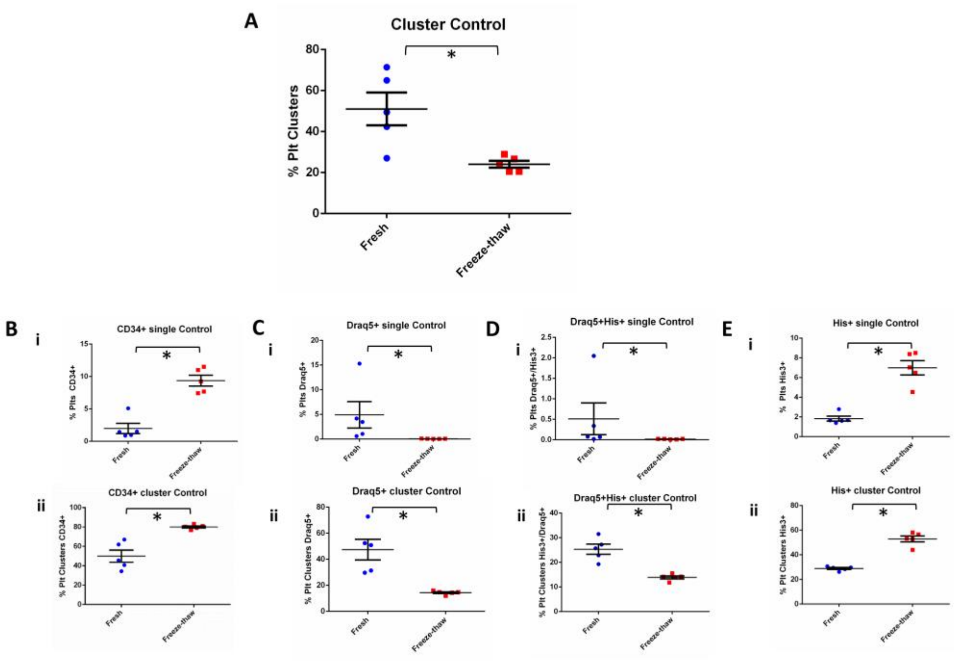

Supplementary Figure 4: Flow cytometry comparison of fresh versus freeze-thawed platelets.

Flow cytometry was performed on both fresh and freeze-thawed platelets, in order to control for the effects of storage conditions on the patient platelet analysis. (A-E) Scatter plots with mean and standard error bars comparing fresh compared to freeze-thawed platelets for both single platelets (i) and clustered platelets (ii). Student unpaired t-test was performed, with significance defined as $p \leq 0.05$ according to a paired student t-test for all samples.

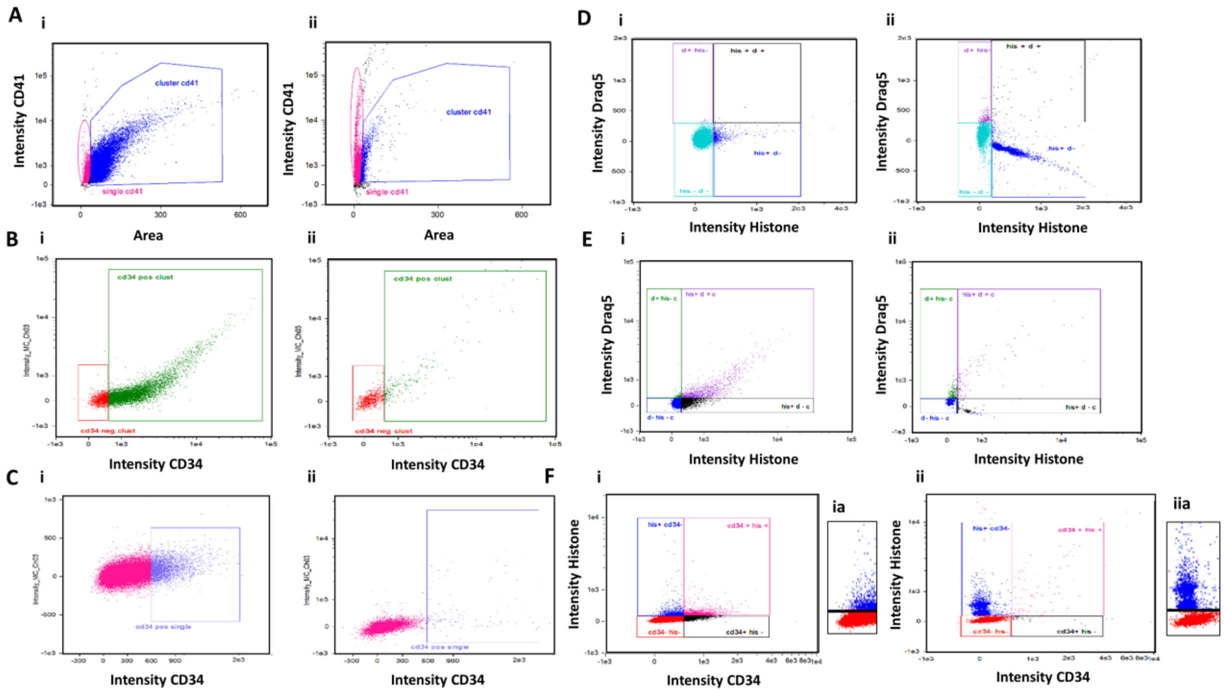

Supplementary Figure 5: Flow cytometry analysis of platelets from patients diagnosed with sepsis.

Flow cytometry was performed on platelets isolated from patients patient with and without sepsis. (A-F) Representative scatter plots of flow cytometry results comparing platelets from control patients (i) and platelets from patients diagnosed with sepsis (ii). (A) CD41 compared to area of cell identifies single versus clusters of CD41 platelets; (B) CD34 intensity in CD41 positive clusters; (C) CD34 intensity in CD41 positive singlets; (D) Intensity of Draq5 and Histone in CD41 positive singlets; (E) Intensity of Draq5 and Histone in CD41 positive clusters; (F) Intensity of CD34 and Histone in CD41 positive singlets. A magnified example of single platelet-associated His3 signal is represented for both the control patient (Fia) and the sepsis patient (Fiia)

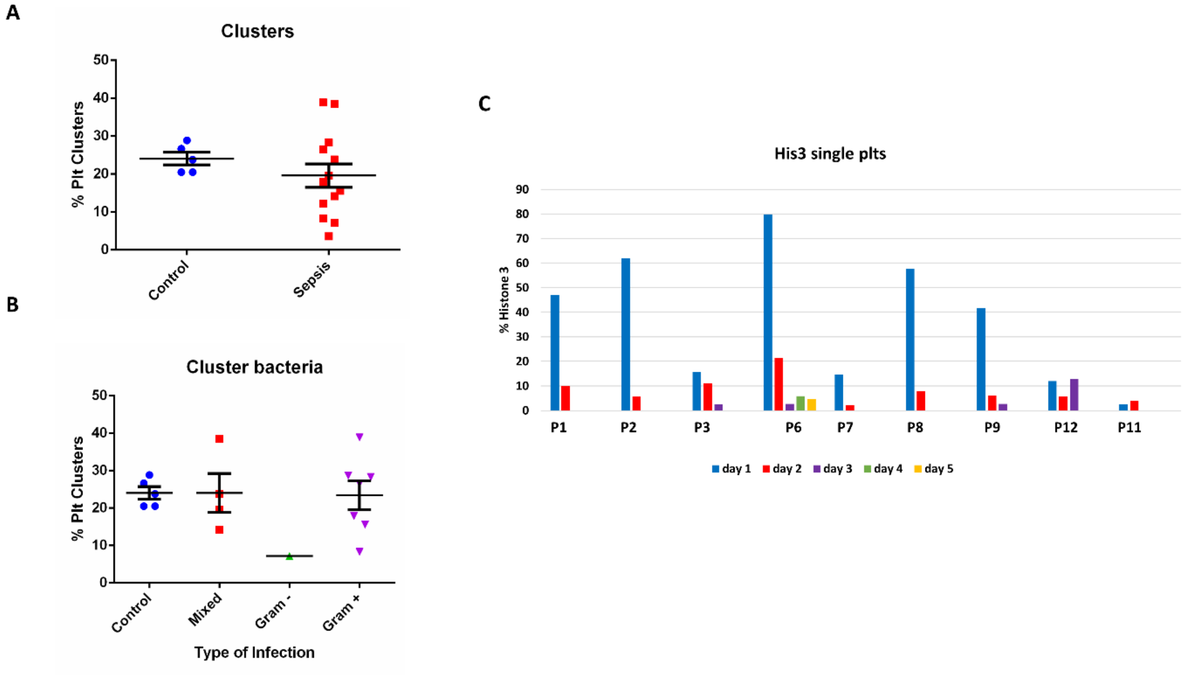

Supplementary Figure 6: Platelet phenotype is associated with the diagnosis of sepsis and the type of infection.

Flow cytometry was performed to evaluate platelet phenotype from patients diagnosed with sepsis. (A) Platelet phenotype was compared for patients with and without sepsis. (B) Platelet phenotype was also compared for patients with different types of infection: Gram +, Gram -, or Mixed. (C) Platelet were evaluated on multiple days of hospitalization. $His3^+$ platelets appeared to be highest in patients with acute sepsis and on day 1 of evaluation and decreased as the patient was treated (P1,2,3,6,7,8,9). Patients with chronic sepsis that had been in the hospital for a prolonged period did not have the same changes in $His3^+$ platelets and these patients did not recover. For A, student t-test was performed and for B, one-way ANOVA testing was performed comparing the type of infection to the control group, with significance being defined as $p < 0.05$

(*). Statistics were not performed on the Gram – group as there was only one patient in this group.

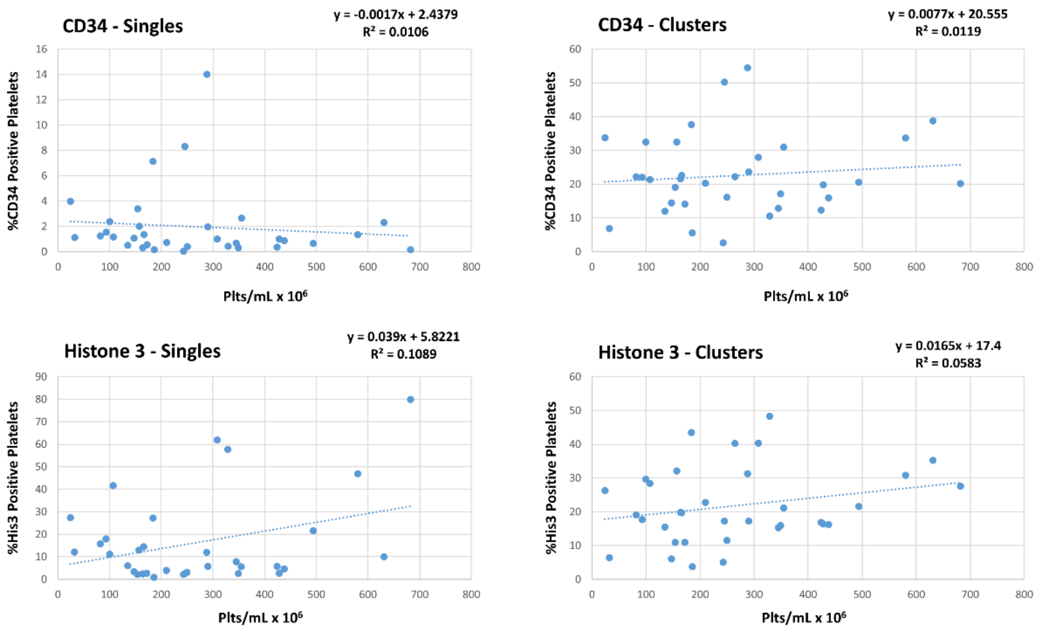

Supplemental Figure 7: Relationship of platelet count and PAH

Flow cytometry of platelets from patients with sepsis were plotted against the platelet count. % CD34 and % Histone 3-positive single platelets (A & B) and % CD34 and % Histone 3-positive platelet clusters showed minimal to no relationship with patient platelet count. Best-fit line (linear) is plotted.

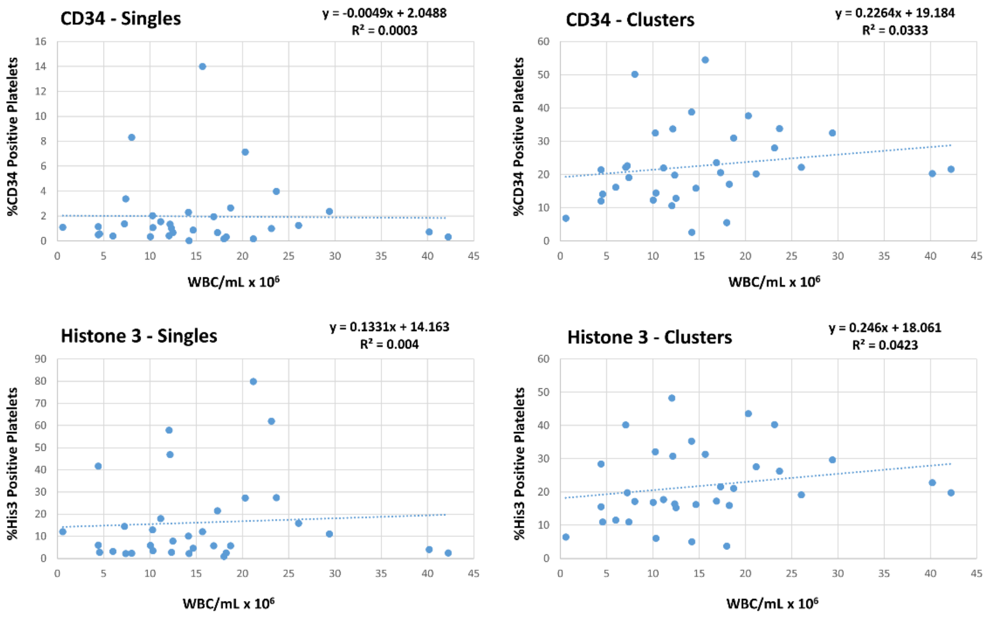

Supplemental Figure 8: Relationship of white blood cell count and PAH

Flow cytometry of platelets from patients with sepsis were plotted against the white blood cell count. % CD34 and % Histone 3-positive single platelets (A & B) and % CD34 and % Histone 3-positive platelet clusters showed minimal to no relationship with patient white blood cell count. Best-fit line (linear) is plotted.

Tables:

Supplementary Table 1: Histone protein measurement.

Histone	MW (kDa)	E/1000
**Histone 2A**	13.960	4.05
**Histone 2B**	13.774	6.07
**Histone 3**	15.273	4.04
**Histone 4**	11.236	5.40

Supplementary Table 2: Histone protein quantification

Sample	Total Protein (OD)[1]	Total Protein[1] (mg/mL)	H2A[2] (mg/mL)	H2B[3] (mg/mL)	H3[4] (mg/mL)	H4[5] (mg/mL)
Calf thymus	0.57[7]	10.35[6]	2.81	1.8	3.3	1.84
Meg-01 cells	2.97[7]	7.07	1.76	1.4	2	1.12
Platelet 1	0.074	0.18	0.27	0.62	0.44	0.18
Platelet 2	0.064	0.15	0.36	0.18	0.34	0.29

1. 230 nm
2. 280 nm; MW (kDa), 13.960; E/1000, 4.05
3. 280 nm; MW (kDa), 13.774; E/1000, 6.07
4. 280 nm; MW (kDa), 15.273; E/1000, 4.04
5. 280 nm; MW (kDa), 11.236; E/1000, 5.40
6. Expected concentration is 10 mg/mL total histones
7. Sample diluted 1:10

Supplementary Table 3: Histone post-translational modifications

Histone Modification	Residue	Modification	Functions
H3k4me3	Lys4	Tri-methylation	Euchromatin, transcriptional activation
H3k56ac	Lys56	Acetylation	DNA damage repair, chromatin assembly
H3s10ph	Ser10	Phosphorlyation	Mitosis, immediate early gene activation
H3k27me3	Lys27	Tri-methylation	Transcriptional silencing
H3s28ph	Ser28	Phosphorylation	Mitosis
Cit H3	N/A	Citrullination	Catalyzed by PAD4
H2A.X	N/A	N/A	DNA damage repair, response to double strand DNA breaks

Supplementary Table 4: Histone post-translational modification quantification

Sample	Total H3[1]	H3k4me3[1]	H3k56ac[1]	H3s10ph[1]	H3k27me3[1]
Positive Control[2] (1:25)	3278.5	3360	2006	2170	2196
Blank	18	9	13	14	10
Meg-01 (1:250)	1760	401	185	296.5	598
Meg-01 (1:500)	1354	175.5	66	108	315
Meg-01 (1:1000)	909	60.5	27	43	151.5
Meg-01 (1:2000)	477	35	16	24	92
Platelet (1:25)	108	23.5	16	15	28
Platelet (1:50)	62	15.5	14	12	21

1. All measurements are provided as MFI (mean fluorescent intensity), with the exception of the LOD row.
2. Positive control of HeLa cell extract, estimated to be 0.5 mg/mL total protein; information provided by Active Motif.

Supplementary Table 5: Histone post-translational modification percentages

Sample	Total H3[1]	H3k4me3[2]	H3k56ac[2]	H3s10ph[2]	H3k27me3[2]
Meg-01 (1:250)	200.0 / 1970.9	22.5	9.9	16.2	33.8
Meg-01 (1:500)	100.0 / 2046.7	12.5	4.0	7.0	22.8
Meg-01 (1:1000)	50.0 / 1995.8	5.8	1.6	3.3	15.9
Meg-01 (1:2000)	25.0 / 1987.4	5.7	0.7	2.2	17.9
Platelet (1:25)	13.75 / 0.46[3]	16.1	3.3	1.1	20.0
Platelet (1:50)	12.75 / 0.42[3]	14.8	2.3	-4.5	25.0
LOD[4]	2	30	7.8	8	3

1. Total input per well in ng / Concentration in units of ng/uL of stock protein
2. % of total H3 based on MFI
3. LOD, Limit of detection. Based on purified histones input. Total ng input.

Supplementary Table 6: Sepsis patient descriptive statistics

Patient ID	Signalment (sex/age)	Infectious Organisms	Source of Infection	Bacterial Classification[1]
1	F/82	- *Klebsiella pneumonia*<br>- Influenza	- Urine	Gram -
2	M/36	- *Pseudomonas aeruginosa*[2]	- Urine	Gram -
3	M/67	- *Enterococcus faecalis*<br>- Yeast<br>- *Staphylococcus aureus*<br>- *Streptococcus pneumoniae*	- Lungs<br>- Lungs<br>- Lungs<br>- Lungs	Gram +
4	M/73	- *Pseudomonas aeruginosa*<br>- *Enterococcus faecium*<br>- Yeast	- Lungs/Urine<br>- Lungs<br>- Lungs	Mixed
5	M/85	- *Enterococcus faecium*<br>- *Candida albicans*<br>- *Coagulase negative Staphylococcus*<br>- Yeast	- Wound<br>- Wound<br>- Lungs<br>- Lungs	Gram +
6	M/49	- *B-haemolytic Streptococcus A*<br>- *Staphylococcus aureus*<br>- *Aspergillus fumigatus*<br>- *Enterococcus faecium*<br>- Influenza	- Lungs<br>- Lungs<br>- Lungs<br>- Lungs	Gram +
7	M/69	- *Streptococcus pneumonia*<br>- Multi-drug resistant *Staphylococcus aureus*<br>- Influenza	- Lungs<br>- Lungs<br>- Blood<br>- Blood	Gram +
8	M/71	- *Enterococcus faecalis*<br>- *Enterobacter aerogenes*<br>- *Corynebacterium*	- Blood<br>- Lungs<br>- Lungs	Mixed
9	M/62	- Multi-drug resistant *Staphylococcus aureus*<br>- *Enterococcus aerogenes*	- Blood<br>- Blood	Gram +
10	M/61	- *Staphylococcus aureus*	- Lungs	Gram +
11	M/77	- *Streptococcus intermedius*	- Pleural fluid	Gram -
12	M/59	- *Klebsiella pneumonia*<br>- *Proteus mirabilis*<br>- *Enterococcus*	- Blood/Lungs<br>- Blood<br>- Blood	Mixed
13	F/76	- Metapneumovirus<br>- No positive bacterial culture	- N/A	N/A
14	F/73	- *Escherichia coli*<br>- *Staphylococcus epidermidis*	- Blood and Urine<br>- Blood	Mixed
15	F/67	- *Escherichia coli*<br>- *Staphylococcus epidermidis*	- Blood/Urine<br>- Urine	Gram +
16	M/81	- *Enterococcus faecalis*	- Blood	Gram -

1. Gram positive, Gram negative or mixed infection (Gram positive and Gram negative bacteria)
2. Culture was negative prior to blood sample collection
